# The Effects of Self-Polymerized Polydopamine Coating on Mechanical Properties of Polylactic Acid (PLA)–Kenaf Fiber (KF) in Fused Deposition Modeling (FDM)

**DOI:** 10.3390/polym15112525

**Published:** 2023-05-30

**Authors:** Sanusi Hamat, Mohamad Ridzwan Ishak, Mohd Sapuan Salit, Noorfaizal Yidris, Syamir Alihan Showkat Ali, Mohd Sabri Hussin, Muhamad Saifuldin Abdul Manan, Muhamad Qauyum Zawawi Ahamad Suffin, Maliki Ibrahim, Ahmad Nabil Mohd Khalil

**Affiliations:** 1 Department of Aerospace Engineering, Faculty of Engineering, Universiti Putra Malaysia, Serdang 43400, Selangor, Malaysia; mohdridzwan@upm.edu.my (M.R.I.);; 2 Faculty of Mechanical Engineering & Technology, Universiti Malaysia Perlis, Ulu Pauh 02600, Perlis, Malaysia; 3 Aerospace Malaysia Research Centre (AMRC), Universiti Putra Malaysia, Serdang 43400, Selangor, Malaysia; 4 Laboratory of Biocomposite Technology, Institute of Tropical Forestry and Forest Products (INTROP), Universiti Putra Malaysia, Serdang 43400, Selangor, Malaysia; sapuan@upm.edu.my; 5 Department of Mechanical and Manufacturing Engineering, Universiti Putra Malaysia, Serdang 43400, Selangor, Malaysia; 6 Advanced Engineering Materials and Composites Research Centre (AEMC), Department of Mechanical and Manufacturing Engineering, Universiti Putra Malaysia, Serdang 43400, Selangor, Malaysia

**Keywords:** FDM, 3D printing, kenaf fiber, polydopamine, PLA, mechanical properties, coating

## Abstract

This research examines the impact of self-polymerized polydopamine (PDA) coating on the mechanical properties and microstructural behavior of polylactic acid (PLA)/kenaf fiber (KF) composites in fused deposition modeling (FDM). A biodegradable FDM model of natural fiber-reinforced composite (NFRC) filaments, coated with dopamine and reinforced with 5 to 20 wt.% bast kenaf fibers, was developed for 3D printing applications. Tensile, compression, and flexural test specimens were 3D printed, and the influence of kenaf fiber content on their mechanical properties was assessed. A comprehensive characterization of the blended pellets and printed composite materials was performed, encompassing chemical, physical, and microscopic analyses. The results demonstrate that the self-polymerized polydopamine coating acted as a coupling agent, enhancing the interfacial adhesion between kenaf fibers and the PLA matrix and leading to improved mechanical properties. An increase in density and porosity was observed in the FDM specimens of the PLA–PDA–KF composites, proportional to their kenaf fiber content. The enhanced bonding between kenaf fiber particles and the PLA matrix contributed to an increase of up to 13.4% for tensile and 15.3% for flexural in the Young’s modulus of PLA–PDA–KF composites and an increase of up to 30% in compressive stress. The incorporation of polydopamine as a coupling agent in the FDM filament composite led to an improvement in tensile, compressive, and flexural stresses and strain at break, surpassing that of pure PLA, while the reinforcement provided by kenaf fibers was enhanced more by delayed crack growth, resulting in a higher strain at break. The self-polymerized polydopamine coatings exhibit remarkable mechanical properties, suggesting their potential as a sustainable material for diverse applications in FDM.

## 1. Introduction

The use of biodegradable materials in 3D printing has become a topic of growing interest as a result of increasing concern for environmental sustainability. Additive manufacturing (AM), also known as 3D printing, has become widely adopted across various industries in the past decade for its capability to produce intricate and customized parts at a low cost using a computer-generated model. The growth of 3D printing has been facilitated by declining costs of 3D printers and the availability of a variety of printing materials [1]. Fused deposition modeling (FDM) is a commonly favored 3D printing technique that uses thermal extrusion to melt and deposit a thermoplastic filament layer by layer, which is then solidified [2]. FDM has been attracting increased attention as a result of its affordability, easy maintenance, and the growing variety of available materials, such as polylactic acid (PLA), polypropylene (PP), polyethylene terephthalate glycol (PETG), and acrylonitrile butadiene styrene (ABS) [3,4,5]. PLA is a biodegradable and eco-friendly linear aliphatic thermoplastic polyester that is derived from renewable sources such as corn or sugarcane fermentation [6]. The use of biodegradable materials like PLA in 3D printing aligns with the growing demand for sustainable solutions and reduces the environmental impact. Ongoing research and development are focused on creating functionalized filaments with improved properties through polymer composites [7,8], with the aim of enhancing the mechanical properties and functionalities of pure polymer parts through the 3D printing of polymer matrix composites [9,10]. Biodegradable filaments made from green composites using PLA and natural fillers [11,12] or fibers [13,14,15] are being developed, offering more advantages, such as a reduced environmental impact as well as low weight and costs [16].

FDM technology-based composites derived from renewable resources have gained appeal in industries such as furniture [17], construction [18], automotive [19], and biomedical [20] and various consumer applications, driven by heightened environmental consciousness and the desire for sustainable materials [21,22]. Recently, the innovation of employing natural fibers as a new sustainable material in polymers for industrial applications has increased significantly, as Khalid et al. reported [23]. In FDM applications, a few natural fibers, such as flax [24], banana fiber [25], wood flour [26], palm oil waste [27], and cocoa shell [28], have also reportedly been used as biodegradable filler materials for reinforcement in the development of filaments for 3D printing. Natural fibers possess desirable attributes, such as cost-effectiveness, low density, elevated strength, and high modulus [29,30]. Composite materials made up of a thermoplastic polymer matrix and a minor proportion of natural fibers are known as natural fiber-reinforced composites (NFRCs) [31]. Although extensive research has been conducted on the mechanical properties of NFRCs produced through traditional methods, such as extrusion and injection molding [31,32], the study of the mechanical behavior of 3D-printed PLA-based NFRCs [33,34,35,36] and the parameters of 3D printing processes, such as infill pattern and geometry [37], is still ongoing.

The kenaf plant, a species of the hibiscus family, is considered a promising option for a biodegradable filler in filaments for 3D printing [38,39]. The plant is highly valued for its fibrous stems, which can be used in a variety of industrial applications, including the manufacturing of paper, textiles, and bioplastics. The structure of the kenaf plant consists of a tall central stem, with lateral branches and leaves growing from nodes along the stem [40,41]. In recent years, kenaf has received growing attention as a sustainable alternative to traditional fiber crops, such as cotton, hemp, and flax. Kenaf is a low-maintenance crop that requires minimal input and has a low environmental impact. Its fast growth rate and high yields make it a promising option for producing large quantities of fiber for industrial applications [42]. Additionally, the fibrous structure of kenaf makes it a potential candidate for use as a natural filler in biodegradable filaments for 3D printing [38,39,43].

The use of kenaf in the production of filaments for 3D printing can improve the mechanical properties of the final printed parts, making it suitable for various applications. The fibrous structure of kenaf allows it to reinforce the polymer matrix, leading to improved tensile strength, impact resistance, and stiffness in the final product [38,39]. Despite its potential, the incorporation of kenaf as a natural filler in filaments for 3D printing is presently at an early stage of development. Research is ongoing to optimize the production process, improve the dispersion of kenaf in the polymer matrix, and enhance the compatibility of the blend [38,44]. The production of kenaf-blended filaments requires a careful balance of processing parameters, such as temperature, extrusion speed, and blending ratio, to achieve the desired properties [38,39,45].

One of the main advantages of using kenaf fiber blended with PLA as a filament for 3D printing is its biodegradability. Unlike traditional 3D printing materials, such as ABS and PETG, which are not biodegradable and can contribute to environmental pollution, kenaf–PLA composite filaments are fully biodegradable and can break down naturally in the environment. However, using kenaf fiber blended with PLA as a filament for 3D printing also presents some challenges. One of the main issues is processing and compatibility difficulties between the two different materials [46,47]. Kenaf bast fibers may not be well dispersed in a PLA matrix, leading to inhomogeneity in the final material and reducing the effectiveness of the reinforcement [38,46,48].

Another drawback is the difficulty in processing the material due to its fibrous nature [49]. Kenaf fibers can cause wear and tear on a 3D printer’s extruder and nozzle, leading to maintenance and downtime issues, such as clogging [50,51]. Additionally, blending kenaf fiber with PLA can make the filament more difficult to print with as a result of its increased viscosity and decreased flowability. This can lead to difficulty achieving a consistent and high-quality print [51,52]. Furthermore, kenaf fiber has a relatively high moisture absorption rate [53], which can affect the dimensional stability of the printed object. A study by Aumnate et al. [51] found that a kenaf–PLA composite had a moisture absorption rate of 3.5% compared to 1% for pure PLA, for which the water contact angle decreased to 63.7° with the addition of the kenaf fiber. Elevated rates of moisture absorption can impact both the dimensional stability and the mechanical attributes of a final printed product [52,54]. However, kenaf fiber cellulose also shows unique properties and characteristics, including its low density and high stiffness and strength that make it highly compatible with PLA, resulting in more homogeneous dispersion texture. This improves the interfacial adhesion strength of the fiber and matrixes [51].

Recently, polydopamine (PDA) has been widely used as a coating for 3D-printed polymers to enhance their surface properties and modulate cell behavior [55]. PDA is a material that is formed by the self-polymerization of dopamine in an alkaline environment. The self-polymerization process is driven by the oxidation of dopamine and the formation of covalent bonds between catechol and amine groups. This results in the formation of a catechol-based polymer network with strong chemical bonds [56,57]. In previous studies by Das and Reches [58] and Cai et al. [59] on the effects of the chemical bonds of dopamine on different surfaces, they found that dopamine produced a good binding structure for connecting peptides, polymers, and other molecules to desired surfaces. Hence, as a result of the unique structure and functional groups of dopamine, PDA can form various types of bonds at PLA matrices and kenaf fiber interfaces, such as the hydrogen bond and coordination bond. These bonds are rather strong and, therefore, could enhance the mechanical properties of the bulk material. The benefits of PDA in polymers such as polylactic acid (PLA) stem from its ability to modify the surface of a substrate and improve its mechanical properties. Recent work by Zhao et al. [55] also found that, by directly coating PLA pellets, the covalent immobilization of PDA on PLA through the PDA anchor effect improves the bonding strength of 3D-printed specimens. PDA has also been shown to improve the adhesion, toughness, and flexibility of polymers, making them more durable, resistant to wear and tear, and better able to withstand mechanical stress [60,61]. In polymer composites, PDA has also been found to increase the hydrophilicity of polymers and natural fibers [62], making them more compatible with aqueous environments [63].

Studies have shown the positive impacts of PDA coating on the mechanical properties, hydrophilicity, and cell behavior of 3D-printed scaffolds. For example, Kao et al. [64] found that PDA–poly(lactic acid) (PLA) scaffolds significantly improved the adhesion, proliferation, and differentiation of human adipose-derived stem cells. Wang [65] observed excellent hydrophilicity and improved mechanical properties in PDA–PLA-coated calcium silicate scaffolds, as well as a positive effect on cell spreading. Furthermore, the study observed that this positive effect on cell spreading was due to the presence of PDA on the surface. A recent study by Tao Chen et al. [66] focused on the potential of 3D-printed scaffolds in the repair of bone defects using polydopamine (HA/CMCS/PDA). The scaffolds demonstrated suitable degradability, good compressive strength, and superior cell attachment performance compared to commercial bone-repair materials. Next, Shiyuan Fan et al. [67] investigated the optimal coating sequence of PDA and CS for BCP scaffolds, finding that the BCP–CS–PDA group displayed the best cell proliferation and osteodifferentiation properties. Both studies contribute valuable insights into the use of polydopamine in the development of 3D-printed scaffolds for bone repair and regeneration.

In conclusion, PDA coating enhances the mechanical properties, hydrophilicity, and cell compatibility of 3D-printed objects [64,65,66,67]. Previous studies by Zhang et al. [63] and Lin et al. [64] suggested that using dopamine as a coating material has a high potential to increase the stiffness of feedstock for 3D printing and probably to address the limitations of natural fiber reinforcement in the polymer composites of biodegradable filaments. As far as the author is aware, there has been no study investigating how the mechanical properties and microstructural behavior of polylactic acid (PLA)–kenaf fiber (KF) composites in fused deposition modeling (FDM) are affected by self-polymerized polydopamine (PDA) coating. The novelty of this research is in the formulation of PDA coating as a coupling agent to improve the binding strength interfaces and enhance the mechanical properties and microstructural behavior of PLA–KF composites in FDM for 3D printing applications. Hence, to overcome the limitations of natural fiber reinforcement, this study blends bast kenaf fiber (5, 10, 15, and 20 wt.%) with PLA and PDA modifications, creating a novel, biodegradable feedstock for 3D printing. In addition to analyzing the microstructural effects of PLA–PDA–KF polymer composite filaments for 3D printing through FTIR and microscopic characterization, this study examines the tensile, compression, and flexural behavior of the printed parts in detail.

## 2. Materials and Methods

In this section, the employed manufacturing process for the fabrication of FDM filaments with PLA–PDA–KF composites is outlined, along with the utilized measurement and analysis methodologies.

### 2.1. Materials

A commercial, biodegradable PLA (Ingeo Biopolymer 3D850) resin with the specific gravity (SG) of 1.24 g/cc, peak melt temperature (PMT) of 180 °C, and melt flow rate (MFR) of 9 g/10 min (210 °C and 2.16 kg) that has a faster crystallization rate and is able to develop improved heat-resistance, developed especially for FFF (fused filament fabrication) applications, was obtained from NatureWorks, Plymouth, MN, USA. A processed bundle of bast kenaf fiber with a diameter range between 50 µm and 80 µm was provided by the National Kenaf and Tobacco Board (LKTN), Perlis, Malaysia. Dopamine hydrochloride (DA) (98%), an ATX tris buffer, dodium periodate $\left({\mathrm{NalO}}_{4}\right)$, and deionized (DI) water was purchased from Sigma Aldrich (Petaling Jaya, Malaysia).

### 2.2. Preparation of PLA-Coated Dopamine and Granule Kenaf Fiber

**Figure 1:**
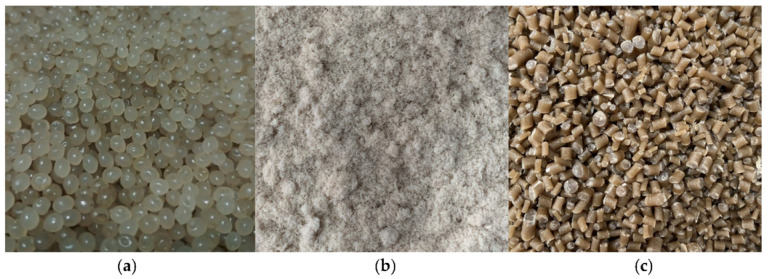
(**a**) PLA pellet coated with dopamine (PLA–PDA), (**b**) granule bast kenaf fiber, and (**c**) PLA–PDA–KF20 (20 wt.%) pellets.

Initially, 2 mg of dopamine hydrochloride was buffered for 2 h with a 10 mM ATX triss until the aqueous solution reached a pH of 8.5, which is typical of marine conditions (PDA). As soon as the PLA pellets were immersed in the dopamine solution, a 4-h stirring procedure was initiated in order to prevent the deposition of PDA nanoparticles produced during the coating process. The finished PLA pellets, coated with dopamine (as shown in Figure 1a), were then rinsed with deionized water and allowed to air dry for an additional four hours at 80 °C [55].

The bundle of kenaf fiber was thoroughly submerged and soaked in distilled water for 4 h to eliminate any dust, debris, and other contaminants. Then, it was left to dry under the sun in the open air for around 8 h and another 2 h in an air-dry oven, BOV-T50F (Biobase, Wolfenbüttel, Germany), at 8 °C to ensure that moisture both inside and outside of the kenaf fiber was eliminated [44,50]. The kenaf fiber was crushed and ground into smaller fragments (<500 µm) using an ultra-fine automatic powder grinder, YF3 (iPharmachine, Wenzhou, China). Given the presence of large particles that could lower the effectiveness of the PLA–kenaf fiber surface interaction in this situation, the kenaf fiber still could not be blended with the PLA. In order to remove the undesired large particles and achieve the granule form of kenaf fiber, a sieving process that employed the automatic sieve shaker machine NL1015X/005 (NL Scientific, Klang, Malaysia) was used. Figure 1b shows the granule form of bast kenaf fiber with an average length of 60 µm.

### 2.3. Preparation of PLA–PDA Pellets Blended with Granule Kenaf Fiber (PLA–PDA–Kenaf Pellet)

The PLA–PDA pellets were dried first in an oven at 80 °C for 6 h to ensure that the moisture inside the pellet was eliminated. This process also helped to reduce the possibility of developing porosity or air burble during the blending process. The PLA–PDA pellets and granule kenaf fiber were then mixed using the plastic mixer machine, WSQB-50 (ACC Ma-chine, Nanjing, China), which rotates at 60 rpm, to produce a uniform blending mixture. To analyze the effects of kenaf fiber on PLA–PDA pellets, 4 weight ratios (wt.%) of PLA–PDA–kenaf have been implemented in this study: 5, 10, 15, and 20 wt.%. Then, the completed mixture of PDA–PLA pellets and granule bast kenaf fiber was added into the twin-screw extruder machine 20 mm (LabTech, Heathfield, UK) with a constant rotation speed of 16 rpm, while the temperature for extrusion was set progressively from the inlet to the head as specified: 170, 175, 180, 185, 190, 190, 190, 190, 190, and 185 °C. The blended PLA–PDA–kenaf composite filament, which had a 3 mm diameter, was converted into pellet form by being chopped in a pelletizer machine until it had a 4 mm length, as illustrated in Figure 1c, which shows the completed blend of the PLA–PDA–kenaf fiber pellet.

### 2.4. Preparation of PLA–PDA–Kenaf Filament

**Figure 2:**
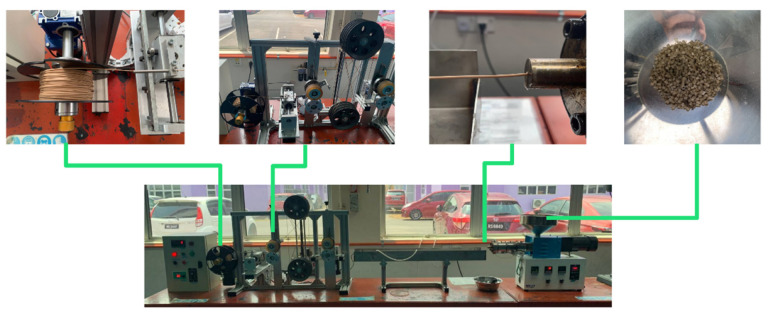
Filament fabrication process for PLA–PDA–KF.

**Figure 3:**
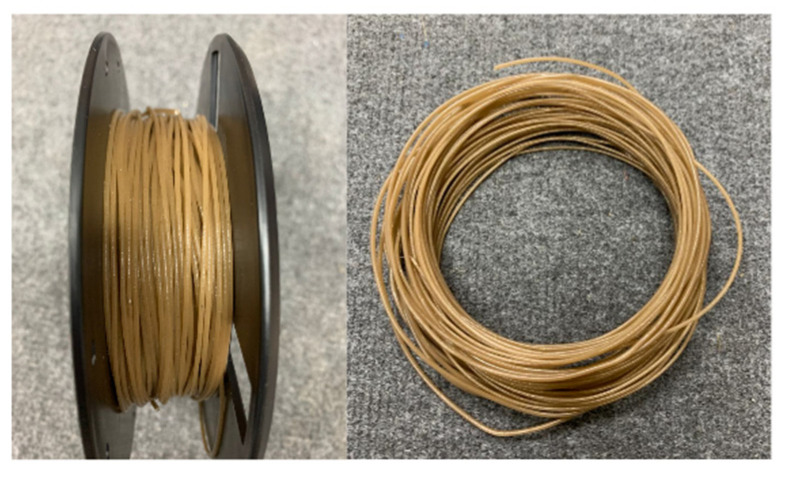
Sample of the 3D printing filament PLA–PDA–KF5 (5 wt.%) with a 1.75 mm average diameter.

**Figure 4:**
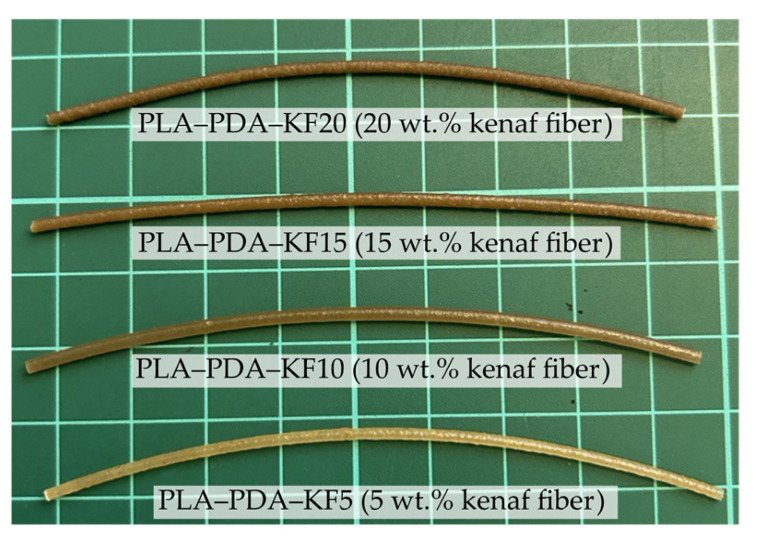
Sample of the 3D printing filament PLA–PDA–KF from different wt.% ratios.

The FDM filament of PLA–PDA–kenaf was produced using the desktop single-screw extrusion machine SJ35 (Robotdigg, Hong Kong, China), as illustrated in Figure 2. This method was carried out using the same commercial filament’s mold diameter (1.75 mm). Based on several experimental attempts and optimization in line with previous research [38,68], the formation of a uniform and stable filament was achieved at three extrusion temperatures from hopper to mold, 120, 175, and 180 °C, with a constant extrusion speed of 5 rpm. Before filament extrusion, the PLA–PDA–kenaf was pre-heated in the air-dry oven BOV-T50F (Biobase, Germany) for 2 h at 60 °C to ensure that the moisture was removed. Next, to ensure that the filament diameters were consistently produced during the extrusion process, an inline filament inspection was performed for every 2 m of length using an inline filament diameter gauge installed in the spooler section [68]. In addition, each fabricated filament sample in the different kenaf fiber ratios was analyzed using SEM to observe the homogenous dispersion of fibers before the process of 3D printing a specimen. Figure 3 shows a sample of the complete 3D printing filament produced from the 5 wt.% kenaf fiber (PLA–PDA–KF5), and Figure 4 shows a sample of the 3D printing filament PLA–PDA–KF fabricated in different wt.% ratios.

### 2.5. Fabrication of PLA–PDA–Kenaf FDM Specimens

**Figure 5:**
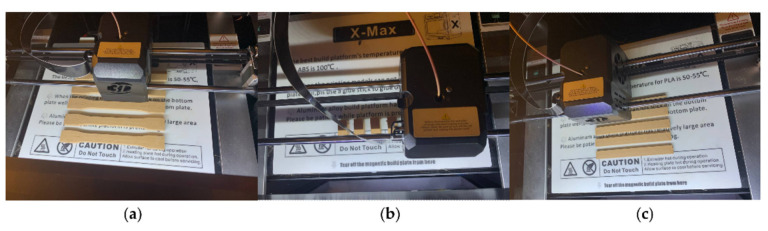
3D-printed specimen design of (**a**) an ASTM D638 (tensile), (**b**) an ASTM D695 (compression), and (**c**) an ASTM D790 (flexural) with a fiber kenaf content of 5 wt.%.

Three types of specimens were designed in the CAD software (CATIA) based on ASTM standards as follows: tensile (ASTM D638) [69], compression (ASTM D695) [70], and flexural (ASTM D790) [71]. The design was imported into the Qidi slicer for G-code generation before being printed using the Qidi X-Max 3D Printer (Wenzhou, China). A line infill pattern with 100% density and a raster angle of 0° and 90° was implemented for all specimens to ensure that the load distribution was in line with the specimen orientation and could, thus, obtain optimum strength during the test [9,16]. Table 1 below shows details of the printing parameters used during specimen fabrication. For each specimen design, five samples were printed according to the standard requirement. Hence, the total number of samples printed based on the four different wt.% ratios (5, 10, 15, and 20 wt.%) and three types of specimen design (ASTM D638, ASTM D695, and ASTM D790) was sixty. Figure 5 shows a sample of the 3D-printed specimen design of (a) an ASTM D638 (tensile), (b) an ASTM D695 (compression), and (c) an ASTM D790 (flexural) with a fiber kenaf content of 5 wt.%.

### 2.6. Characterization

#### 2.6.1. FTIR on PLA–PDA-Coated Pellets

An FTIR analysis was performed on virgin PLA (3D850), PLA–PDA, and PLA–PDA–kenaf pellets. The pellets, which were less than 2 mm in diameter and 3 mm in length, were analyzed using Spectrum 3 (PerkinElmer, Waltham, MA, USA) in transmission mode. The scanning range was from 4000 cm^−1^ to 400 cm^−1^ with a resolution of 4 cm^−1^. To eliminate any possible errors, each pellet was measured three times.

#### 2.6.2. Mechanical Characterization of PLA–PDA–Kenaf FDM-Printed Specimens

**Figure 6:**
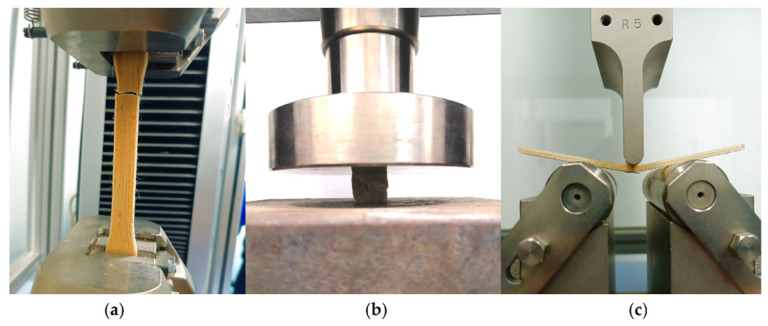
Mechanics of 3D-printed specimen during the test of (**a**) an ASTM D638 (tensile), (**b**) an ASTM D695 (compression), and (**c**) an ASTM D790 (flexural) with a fiber kenaf content of 5 wt.%.

Tensile, compression, and flexural tests were conducted using the MODEL AG-X 50 kN (SHIMADZU, Tokyo, Japan). The dimensions of the tensile test specimen, which was based on ASTM D638, were 165 × 20 × 4 mm, with the central part of the specimen’s width being 13 mm. A crosshead speed rate of 1 mm per minute was used to test five specimens. Figure 6a depicts the 3D-printed PLA–PDA–KF5 mechanical specimen being put through a tensile test.

Figure 6b shows a sample 3D-printed specimen of PLA–PDA–KF5 mechanical during a compression test. The compression test specimen, according to ASTM D695, had dimensions of 20 × 20 × 40 mm and was measured using the same tensile test crosshead speed rate of 1 mm/min for five specimens.

The density of PLA–PDA–KF in different wt.% was calculated using Equation (1):

(1) $$\rho_{c}=\Sigma {\left(wt.\;\times \rho \right)}_{i+1}$$

where $\rho_{c}$ is the density of the FDM composite filament (kg/m³), *wt.* is the mass content of the matrix and fiber (kg), and ρ is the density of the matrix and fiber (kg/m³).

The tensile and compressive strength of the FDM specimen of PLA–PDA–KF was calculated using Equation (2):

(2) $$\sigma =\frac{F}{A}$$

where *σ* is the tensile and compressive strength of the FDM specimen (Pa), *F* is the maximum force at break (N), and *A* is the area of the cross-section (m^2^).

The modulus of resilience of the FDM specimen of PLA–PDA–KF was calculated using Equation (3):

(3) $$U_{𝓇}=\frac{\sigma_{el}^{2}}{2E}$$

where $U_{𝓇}$ is the modulus of resilience of the FDM specimen of PLA–PDA–KF (MJ/m³), $\sigma_{el}$ is the stress at the elastic limit (Pa), and *E* is the elastic modulus (Pa).

The modulus of the toughness of the FDM specimen of PLA–PDA–KF was found using Equation (4):

(4) $$U_{𝓉}=\left(\frac{\sigma_{el}+\sigma_{uts}}{2}\right)\cdot \varepsilon_{u}-{\left(\frac{\sigma_{el}+\sigma_{uts}}{2}\right)}^{2}\cdot \frac{1}{2E}$$

where $U_{𝓉}$ is the modulus of the toughness of the FDM specimen of PLA–PDA–KF (MJ/m³), $\sigma_{el}$ is the stress at the elastic limit (Pa), $\sigma_{uts}$ is the ultimate tensile stress (Pa), $\varepsilon_{u}$ is the ultimate strain (%), and *E* is the elastic modulus (Pa).

Figure 6c illustrates the flexural testing process of a 3D-printed specimen made from PLA–PDA–KF5 with a sample dimensions of 80 × 20 × 5 mm. All five specimens likewise used the identical crosshead speed rate of 1 mm/min. Every test was carried out at room temperature. 

The flexural strength of the FDM specimen of PLA–PDA–KF is calculated using Equation (5):

(5) $$\sigma =\frac{3PL}{2bd^{2}}$$

where *σ* is the flexural strength of the FDM specimen (Pa), *P* is the maximum force at break (N), *L* is the support span (mm), *b* is the width of the beam tested (mm), and *d* is the depth of the beam tested (mm).

#### 2.6.3. Microstructure Morphology Analysis (SEM)

**Figure 7:**
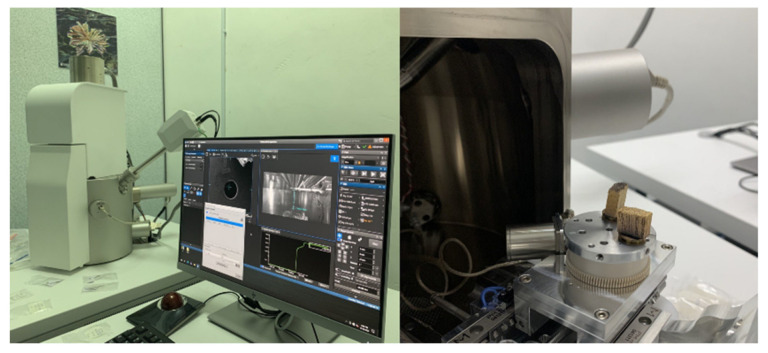
Microstructure morphology analysis (SEM) of 3D printed ASTM D638 specimens of PLA–PDA–KF5.

An SEM VEGA 4 (Vega Compact, Tescan, Warrendale, PA, USA) with an acceleration voltage of 10 kV was used to observe the microstructure of the PDA coating on the pellets and the mechanical tests of the tensile, compression, and flexural fracture surface. Before analysis, all specimen types were given a 10 nm (30 s) layer of spray gold to enable the coated surface to be more conductive. Figure 7 shows the SEM analysis of the specimen sample of the 3D printed ASTM D638 of PLA–PDA–KF5.

## 3. Results and Discussions

### 3.1. FTIR Analyses of PDA–PLA Coated Pellets

**Figure 8:**
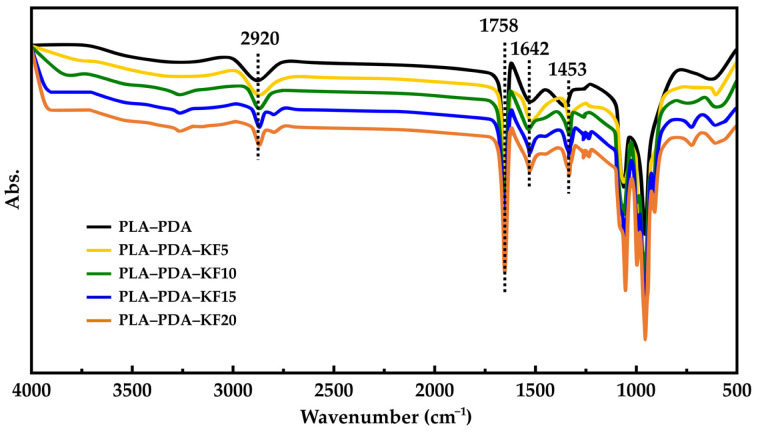
FTIR spectra of dopamine coating on virgin PLA and PLA–kenaf.

The FTIR spectra of the virgin PDA–PLA and PDA–PLA–kenaf fiber (5, 10, 15, 20 wt.% of kenaf fiber) are presented in Figure 8.

Upon examining Figure 8, we observe the spectral features of both virgin PLA–PDA and PLA–PDA–kenaf pellets. The broad OH stretching bands, indicative of polymeric hydrogen bonding, fall within the 3300–3100 cm**^−^**^1^ range. Aromatics’ C-H stretching bands emerge at 2920 cm**^−^**^1^, while N-H bending bands are seen at 1642 cm**^−^**^1^. The stretching vibration of C=O in ester groups and the asymmetric deformation of C–H in CH3 correspond to the spectral peaks at 1758 and 1453 cm^−1^, respectively. In comparison to unmodified PLA pellets, PLA–PDA pellets exhibit an overlapping peak at 1642 cm**^−^**^1^, indicating C=C resonance vibrations in the aromatic ring and N–H bending vibrations, and a weak peak at 1410 cm^−1^, indicating N–H shearing vibrations [62]. These novel peaks signify a robust dopamine-coating reaction on the surfaces of both virgin PLA and PLA–kenaf pellets. A subtle distinction in the five spectra is noted at 2920 cm**^−^**^1^: the PLA–PDA–KF20’s characteristic peak intensity is significantly diminished, suggesting that dopamine’s phenolic hydroxyl and amino groups form hydrogen bonds with the kenaf fiber’s surface free hydroxyl groups. The characteristic peak intensity decreases progressively for PLA–PDA and PDA–PLA–KF5 through to PDA–PLA–KF20, implying increased cellulose exposure to dopamine treatment as well as an augmented specific surface area and enhanced kenaf fiber polarity. Concurrently, dopamine treatment falls short of achieving dopamine coating under the same conditions as PLA–PDA, explaining the stronger characteristic peak of lesser wt.% kenaf fiber. The lowest characteristic peak intensity of PLA–PDA–KF20, at 3415 cm**^−^**^1^, is due to the reaction between alkali and cellulose, with an increasing alkali concentration leading to sodium cellulose formation and subsequent kenaf fiber surface breakage. The carbonyl (C=O) stretching vibration peaks, visible in all five spectra at 1758 cm**^−^**^1^, vanish post-dopamine treatment. It has been reported that dopamine solutions dissolve specific hemicellulose groups within the carbonyl absorption peak region [49]. Hence, applying dopamine coating can remove impurities such as wax, lignin, and hemicellulose from kenaf fiber while it is being blended. By melting PLA and dopamine at the extrusion temperature, kenaf fiber can be coated and treated, leading to improved interfacial properties in the resulting composites.

### 3.2. The Effects of PDA Coating and Kenaf Fiber on Mechanical Properties of PLA–Kenaf Composite FDM-Printed Specimens

**Figure 9:**
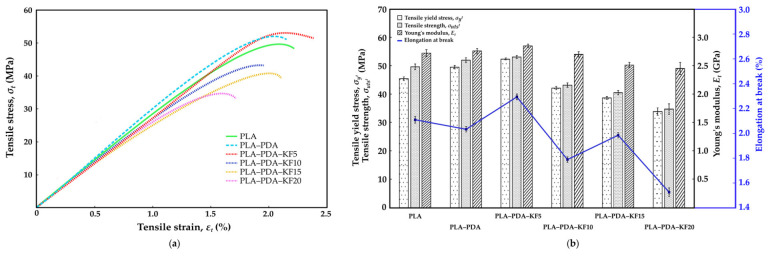
(**a**) Tensile stress–strain curve; (**b**) mechanical properties of tensile test.

**Figure 10:**
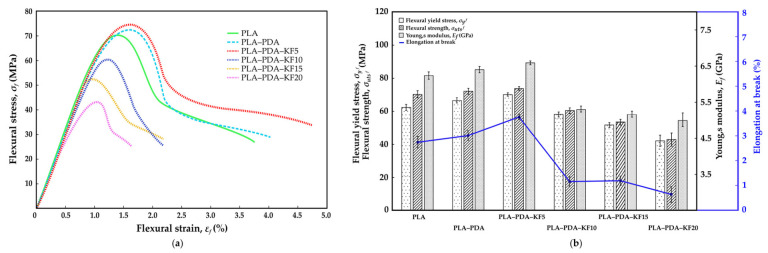
(**a**) Flexural stress–strain curve; (**b**) mechanical properties of flexural test.

**Figure 11:**
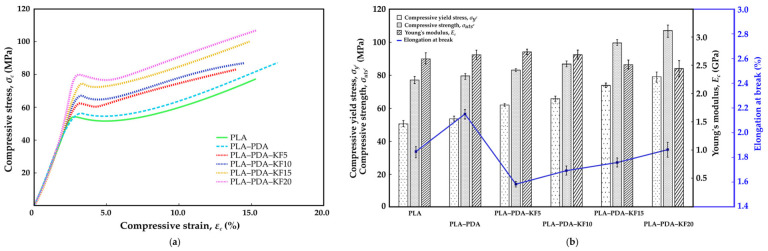
(**a**) Compression stress–strain curve; (**b**) mechanical properties of compression test.

Figure 9a, Figure 10a and Figure 11a show the stress–strain curve of the performed tensile, flexural, and compression tests, respectively, for a virgin PLA, PLA-coated PDA (PLA–PDA), and PLA-coated PDA with a kenaf fiber blend at four different weight percentages (wt.%): 5 wt.% (PLA–PDA–KF5), 10 wt.% (PLA–PDA–KF10), 15 wt.% (PLA–PDA–KF15), and 20 wt.% (PLA–PDA–KF20). Figure 9b, Figure 10b and Figure 11b summarize all of the mechanical data from the tensile, flexural, and compression tests in Figure 9a, Figure 10a and Figure 11a, respectively. Most of the specimens coated with PDA and combined with kenaf fiber that underwent tensile testing showed the typical curve form of virgin PLA. For all PLA–PDA–KF specimens, the typical initial linear elastic behavior was shown at more than 35 MPa; beyond that, a nonlinear zone was seen up until the point of maximum stress. Upon reaching the maximum stress peak, a decrease in stress was observed with an increase in strain until the system eventually failed.

The maximum tensile stress was achieved by PLA–PDA–KF5 at about 54.6 MPa (Figure 9a), with the highest strain of 2.45%, followed by PLA–PDA at 52.3 MPa and virgin PLA, which is only 49.7 MPa [48]. The improvement in tensile properties with the addition of kenaf fiber and PLA–PDA coating is due to an enhanced load-bearing capacity and interfacial adhesion, as well as the homogeneous dispersion of the fiber in the matrix. The optimum fiber content for maximum tensile strength is 5 wt.%, while higher fiber content can result in a decrease in mechanical properties. Furthermore, as shown in Figure 9b, the Young’s modulus of the PLA coated with PDA blended with kenaf fiber (PLA–PDA–KF) is still in range of the virgin PLA’s average Young’s modulus level, between 2.47 GPa and 2.86 GPa. This trend was similar to the flexural results, where PLA–PDA–KF5 produced the highest flexural stress of 75.2 MPa (Figure 10a) and produced an average Young’s modulus of 6.56 GPa (Figure 10b) while being capable of extending the strain to 4.74% before break [54]. This indicates that the addition of kenaf fiber to the PLA–PDA blend improved its yield strength in tensile and flexural tests by approximately 13.4% and 15.3%, respectively, compared to virgin PLA [72] for the maximum value produced by PLA–PDA–KF5 3D-printed specimens. Moreover, the tensile strength and flexural performance derived from the PLA–PDA–KF5 composite have demonstrated a substantial improvement when juxtaposed with the preceding research conducted by Lau et al. [38], which only employed a blend of PLA–KF at the same concentration of 5 wt.%. This marked enhancement may be attributed to the elevated reactivity inherent in polydopamine (PDA), which favorably expedites the creation of chemical bonds within a PLA matrix [56,58,59,64].

Furthermore, PDA acts as an additional reflection platform, thereby augmenting the interfacial adhesion between the fibers and the matrix of the PLA–KF composite. As a result, there is an efficient transfer of stress between the fibers and the matrices [73]. However, the addition of higher percentages of kenaf fiber did not significantly improve the strength, as demonstrated by the similar stress–strain curves observed for PLA–PDA–KF10, PLA–PDA–KF15, and PLA–PDA–KF20 in both tensile and flexural tests as shown in Figure 9a and Figure 10a. These findings only demonstrate a reduced yield in stress and strain from virgin PLA, which are roughly 25.5% and 32.1% lower, respectively, in collecting data from PLA–PDA–KF20 3D-printed specimens. This decrease in tensile and flexural mechanical properties could be explained by the fact that adding more kenaf fiber to PLA than 5 wt.% causes particle agglomeration and voids. This was partly attributed to the poor adhesion between the surface of the PLA matrix and that of the kenaf fiber during tension and shearlike conditions, which decreased the material’s stiffness [29,38,54]. However, the right ratio of KF in PLA–PDA should provide a higher strength in the composite structure, as the combination of PLA–PDA–KF5 has acquired the maximum tensile stress properties. The real application of PLA–PDA–KF polymer composites would be in the furniture industry, where they customize modular joint components between main furniture parts. The modular joint components would be a woodlike texture that would render the furniture similar to wood.

Nevertheless, adding additional kenaf fiber to PLA–PDA has significantly increased its compressive stress. According to Figure 11a, the PLA–PDA–KF20 produced a compressive stress of up to 108.6 MPa, which is nearly 30% higher than the 78.8 MPa of virgin PLA, and completely fractured at a strain of 15.4% [37] while obtaining the lowest Young’s modulus (Figure 11b) in comparison to virgin PLA. The PDA treatment of PLA, which removes impurities and improves porosity on the surface of kenaf fiber, is the main cause of this. This leads to a more compact structure during compression and better compressive capabilities. In order to minimize damage, kenaf fiber also aids in concentrating stress at the fracture tip during load compression [47,73]. The polymerization effect of PDA facilitates the infiltration of PLA into the deeper layers of kenaf fiber, enhancing the physical combination of PLA–PDA and kenaf fiber. The results suggest that the addition of kenaf fiber to the PLA–PDA blend can improve its mechanical properties, but there may be a limit to the amount of fiber that can be added before the benefits plateau. The stress–strain curve provides a clear picture of the behavior and pattern of the material under tension, flexural, and compression tests. The addition of polydopamine (PDA) and kenaf fiber (KF) to polylactic acid (PLA) results in a complex interplay of mechanical properties, as demonstrated by the increase mostly in compression, flexural and tensile strength.

In addition, the elucidation of the kenaf fiber effects in tensile mechanical properties can also be observed in terms of density based on kenaf fiber wt.%. This provides more precise information regarding the effectiveness of the mechanical tensile strength. Hence, the density of the PLA–PDA–KF composite was calculated using the weight fraction of kenaf fiber in the PLA matrix. PLA (3D850) had a density of 1.24 g/cm^3^ [74], while that of kenaf fiber in granulate form was 0.12 g/cm^3^ [53]. Table 2 and Table 3 show the tensile mechanical properties of PLA, PLA–PDA, and PLA–PDA–KF FDM composites.

Upon analyzing the data presented in Table 2, it can be observed that the incorporation of PDA into PLA (PLA–PDA) results in an enhancement of both specific modulus and specific tensile yield strength by approximately 9.9% and 17.5%, respectively. The further addition of KF at different percentages (5%, 10%, 15%, and 20%) influences these properties in various ways. The PLA–PDA–KF5 specimen demonstrates the highest specific tensile yield strength, which is 36.5% higher than that of pure PLA. This improvement can be attributed to the effective stress distribution as a result of enhanced fiber–matrix interfacial adhesion, which results from the reactivity of PDA [60,66]. However, as the KF content increases (from PLA–PDA–KF10 to PLA–PDA–KF20), there is a decline in specific tensile yield strength, possibly as a result of a saturation effect in the microstructure, which leads to poor stress distribution and fiber agglomeration [29,38,54]. Despite these reductions in the tensile mechanical properties, the specific Young’s modulus showed an upward trend as the kenaf content increased, and the specific tensile yield strength appeared to level off to some extent. The low density of KF, due to its closed cell morphology, contributed to a decrease in the density of the composite as the kenaf content increased, resulting in an increase in the specific properties, defined as the ratio of the measured properties to the density of the composite. In terms of the specific modulus, PLA–PDA–KF10 exhibits the highest value, which is around 5.5% higher than that of pure PLA. The increase in KF content from 5 wt.% to 10 wt.% appears to have a positive impact on the specific modulus. 

However, further increasing the KF content (from PLA–PDA–KF15 to PLA–PDA–KF20) causes a decrease in the specific modulus, which may be due to insufficient fiber–matrix adhesion and a less homogeneous distribution of fibers in the matrix [38,50]. Table 3 displays the modulus of resilience, modulus of toughness, and fracture strain for the different specimens. The addition of PDA and KF to PLA generally leads to improvements in these properties. The highest modulus of resilience and toughness are observed in PLA–PDA–KF5, with increases of approximately 36.5% and 13.3%, respectively, compared to pure PLA. These enhancements can be attributed to the increased fiber–matrix interfacial adhesion, stress distribution, and reactivity of PDA with both the matrix and the fibers similar to previous observations [38,39,50,54]. In terms of fracture strains, the PLA–PDA–KF15 specimen exhibits the highest value, approximately 107.7% higher than that of pure PLA. This improvement is likely due to the increased fiber–matrix adhesion and stress distribution, which allows the composite material to withstand higher deformation before failure.

### 3.3. The Effects of PDA Coating and Kenaf Fiber on Fracture Surfaces of PLA–Kenaf Composite FDM-Printed Specimens

**Figure 12:**
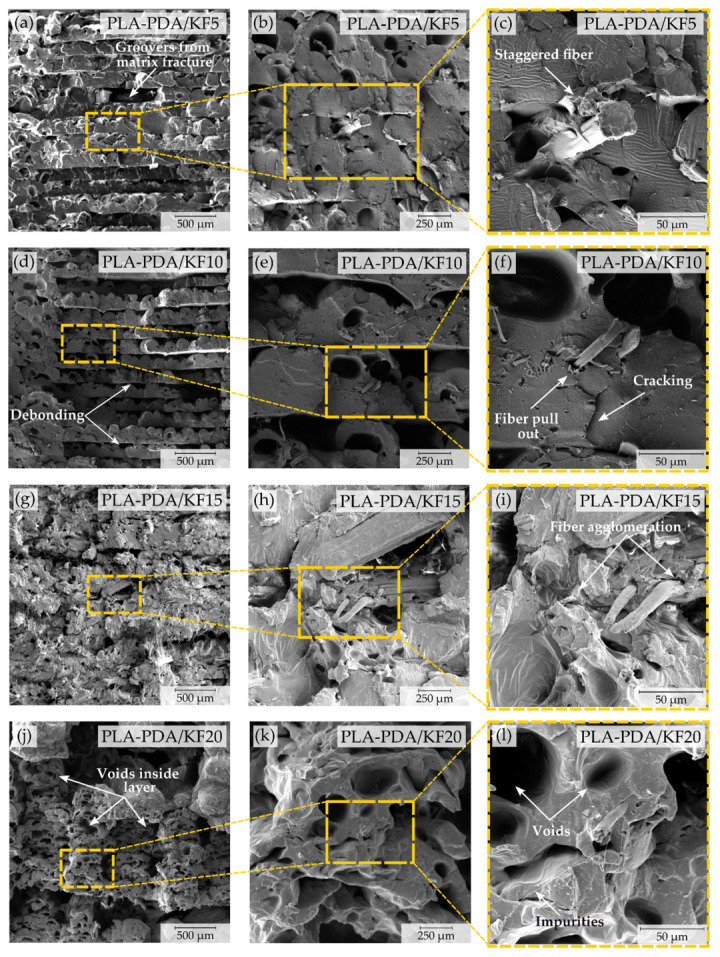
SEM images of the fracture surface after tensile testing of the 3D-printed PLA–PDA–KF: (**a**) PLA–PDA–KF5 (500 µm); (**b**) PLA–PDA–KF5 (250 µm); (**c**) PLA–PDA–KF5 (50 µm); (**d**) PLA–PDA–KF10 (500 µm); (**e**) PLA–PDA–KF10 (250 µm); (**f**) PLA–PDA–KF10 (50 µm); (**g**) PLA–PDA–KF15 (500 µm); (**h**) PLA–PDA–KF15 (250 µm); (**i**) PLA–PDA–KF15 (50 µm); (**j**) PLA–PDA–KF20 (500 µm); (**k**) PLA–PDA–KF20 (250 µm); and (**l**) PLA–PDA–KF20 (50 µm).

The cross-sectional fracture surface morphologies of the 3D-printed PDA-PLA specimens with varying kenaf fiber weight percentages are presented in Figure 12. The mechanical properties of these composites, as influenced by the different kenaf fiber reinforcement approaches, can be elucidated by examining their interfacial characteristics. Figure 12a depicts a relatively smooth surface with minimal gaps and voids for the PLA–PDA–KF5 sample, signifying that dopamine enhances the interfacial adhesion between the kenaf fiber and the PLA matrix. However, matrix fracture grooves in the bed layer are observed as a result of tensile failure. A flaw in the kenaf fiber’s structure during the grinding process is indicated by the staggered fibers in Figure 12c. Nevertheless, the fiber–PLA matrix interaction exhibits good contact, indicating a more uniform bonding of the kenaf fiber’s surface, attributable to the PDA-coating elements. The microstructure of the PLA–PDA–KF10 sample, as displayed in Figure 12d, reveals prominent interlayer debonding between neighboring bead-printing layers. This debonding can be attributed to suboptimal bonding formed during the fused deposition modeling (FDM) process, as well as stress concentrations originating from pre-existing voids that developed during FDM. Comparable debonding incidents have been reported in previous research on printed PLA specimens [9,45,72]. Potential contributing factors to this debonding include the presence of voids and bead shrinkage, which are consequences of the semi-crystalline characteristics of PLA [51]. These factors could lead to inadequate surface contact between adjacent beads [50], resulting in compromised inter-bead bond strength. Consequently, when subjected to external loads, these weakened bonds between neighboring beads may ultimately succumb to debonding, impacting the overall mechanical performance of the specimens. 

Moreover, as illustrated in Figure 12f, fiber bundles in the material’s cross-section are pulled out, accompanied by cracking lines and voids, suggesting suboptimal compatibility between kenaf fibers and the polylactic acid matrix [26,54]. Nonetheless, the matrix’s grain surface remains smooth as a result of PDA-induced chemical reactions. A comparison between PLA–PDA–KF15 (15 wt.%) and PLA–PDA–KF20 (20 wt.%) samples reveals a more pronounced presence of micro defects. A distinct henequen fiber agglomeration in the specimen’s cross-section, seen in Figure 12i for PLA–PDA–KF15, is more significant than in the PLA–PDA–KF5 and PLA–PDA–KF10 samples. Voids and holes within layers appear larger and more frequent in the PLA–PDA–KF20 sample, as illustrated in Figure 12j. This phenomenon can be attributed to an increased kenaf fiber content in the PLA matrix, leading to chemical incompatibility between the hydrophobic thermoplastic molecules and the hydrophilic lignocellulosic molecules of natural fibers. Moreover, a higher kenaf fiber content makes a specimen more porous and bigger voids as seen detail in Figure 12k,l, which causes longer fiber aggregation under loading conditions. Additionally, uncertain dopamine concentration levels may cause a dopamine-modified surface coating to become inadequate for accommodating the cellulose-specific surface area’s sudden increase under identical conditions. Consequently, the composite’s properties deteriorate, and the kenaf fiber undergoes irregular rupturing, indicating that the sodium hydroxide has compromised the kenaf fiber’s structure [24,38,39,55]. This degradation ultimately leads to the loss of mechanical properties in samples with a higher kenaf fiber weight percentage. To summarize, modifying the surface of PLA coated with PDA has a significant effect on the way the fiber interacts with the PLA matrix. By optimizing the dopamine modification, it is possible to improve the overall properties of the composites.

## 4. Conclusions

A biodegradable, natural, fiber-reinforced composite (NFRC) for FDM filaments was developed by coating PLA with dopamine and reinforcing it with 5–20 wt.% bast kenaf fibers. These filaments were used to 3D print tensile, compression, and flexural test specimens at 100% infill density with a raster angle of 0° degrees. Chemical, physical, and microscopic characterizations of blended pellets and FDM-printed composite materials were performed, and the effects of polydopamine coating as well as bast kenaf fiber content on mechanical properties were investigated.

Our findings led to the following conclusions:

▪ By adding dopamine to the filament composites (PLA–PDA–KF), it acted as a coupling agent that created chemical bonds between kenaf fibers and the PLA matrix. This led to an improvement in the interfacial adhesion of PLA and KF, leading to improved tensile, compression, and flexural properties.
▪ As the content of bast kenaf fiber increased, there was a proportional increase in the density and porosity of the PLA–PDA–KF FDM specimens.
▪ The increase in the Young’s modulus observed in PLA–PDA–KF5 can be attributed to the stable condition of optimum homogeneity in the blended composite, which can enhance bonding between kenaf fiber particles and the PLA matrix, along with the decrease in clustering and empty spaces compared to the initial printed PLA.
▪ The tensile, compressive, and flexural stresses and strains at break of blended FDM filament composites with up to 5% kenaf fiber content surpassed those of the virgin printed PLA as a result of the reinforcement provided by kenaf fibers, which delayed crack growth. However, for contents of 5% and higher, the tensile, compressive, and flexural stresses of the FDM-printed specimen were lower than the virgin printed PLA.
▪ Upon conducting a tensile test on the FDM specimen of the PLA–PDA–KF composite, it was observed through microscopic characterization that an escalation in voids and defects occurred as the kenaf content increased. This phenomenon was attributed to the agglomeration of particles within the material.
▪ Based on the results, FDM-printed composites made from PLA-coated PDA with kenaf fiber reinforcement possess remarkable mechanical properties, indicating their potential to be utilized as sustainable materials. The addition of dopamine as an adhesion agent during pellet coating further strengthens the printed FDM composite compared to regular PLA–KF blending, increasing the interfacial strength between the kenaf fiber surface and PLA matrix.

In conclusion, this study highlights the potential of using dopamine-coated PLA with kenaf fiber in the development of sustainable and mechanically improved NFRCs for FDM applications. This research shows that the interfacial bonding induced by PDA helps improve mechanical performance. To fully comprehend the mechanical performance of these materials, more investigation is needed on the binding mode and strength of the interfacial bonds and the structure of interfaces between the various type of polymers and fibers.

## Figures and Tables

**Table 1:** Printing parameters (Qidi X-Max printers) for specimen fabrication.

Parameters	Value
Printing speed	40 m/s
Layer height	0.2 mm
Nozzle diameter	0.4 mm
Extruder temperature	210 °C
Bed temperature	55 °C
Infill pattern	Line
Infill density	100%
Raster angle	0° or 90°

**Table 2:** Density and tensile mechanical properties (specific modulus, specific tensile yield strength) of PLA, PLA–PDA, and PLA–PDA–KF FDM composites.

Specimen	Density (kg/m³)	Specific Modulus MPa/(kg/m³)	Specific Tensile Yield Strength Pa/(kg/m³)
PLA	1240 ^ab^	2.2 (±0.059) ^c^	41.6 (±0.681) ^bc^
PLA–PDA	1253 ^a^	2.4 (±0.050) ^a^	48.9 (±0.580) ^b^
PLA–PDA–KF5	1197 ^c^	2.3 (±0.030) ^b^	56.8 (±0.343) ^a^
PLA–PDA–KF10	1166 ^c^	2.3 (±0.049) ^b^	33.5 (±0.561) ^c^
PLA–PDA–KF15	1122 ^cd^	2.2 (±0.047) ^c^	39.1 (±0.542) ^c^
PLA–PDA–KF20	1079 ^d^	2.3 (±0.108) ^b^	27.8 (±1.250) ^d^

Note: The data in Table 2 were evaluated using a one-way ANOVA with a 95% confidence threshold. Standard deviations for the test outcomes are documented within brackets, while alphabetical indicators represent statistical disparities. When groups share identical letter(s), they exhibit no substantial distinction from each other, and the opposite holds true.

**Table 3:** Tensile mechanical properties (modulus of resilience, modulus of toughness, and fracture strain) of PLA, PLA–PDA, and PLA–PDA–KF FDM composites.

Specimen	Modulus of Resilience (MJ/m³)	Modulus of Toughness (MJ/m³)	Fracture Strain (%)
PLA	41.6 (±0.168) ^b^	65.5 (±0.168) ^b^	0.26 (±0.230) ^d^
PLA–PDA	48.9 (±0.143) ^ab^	65.9 (±0.143) ^ab^	0.33 (±0.196) ^c^
PLA–PDA–KF5	56.8 (±0.085) ^a^	74.2 (±0.085) ^a^	0.51 (±0.116) ^b^
PLA–PDA–KF10	33.5 (±0.138) ^cd^	48.6 (±0.138) ^cd^	0.35 (±0.189) ^c^
PLA–PDA–KF15	39.1 (±0.134) ^c^	51.3 (±0.134) ^c^	0.54 (±0.183) ^a^
PLA–PDA–KF20	27.8 (±0.309) ^d^	35.8 (±0.309) ^d^	0.36 (±0.320) ^c^

Note: The data in Table 3 were evaluated using a one-way ANOVA with a 95% confidence threshold. Standard deviations for the test outcomes are documented within brackets, while alphabetical indicators represent statistical disparities. When groups share identical letter(s), they exhibit no substantial distinction from each other, and the opposite holds true.

## Data Availability

The data presented in this study are available on request from the corresponding author. The data are not publicly available as a result of on-going relevant study.
